# Recent Advances in Exosome-Based Therapeutic Strategies for Acute Lung Injury: Mechanisms and Translational Advances

**DOI:** 10.3390/antiox15050617

**Published:** 2026-05-13

**Authors:** Joon-Ha Song, Hye-Ryun Kim, Dong-Ha Song, Su-Min Jin, Won-Jae Ko, Jinbong Park, Ki-Eun Hwang, Yohan Han

**Affiliations:** 1 Department of Microbiology, Wonkwang University School of Medicine, Wonkwang University, 460 Iksandae-ro, Iksan 54538, Jeonbuk, Republic of Korea; ssjh527@wku.ac.kr (J.-H.S.); gpfus@wku.ac.kr (H.-R.K.); sdh12933@wku.ac.kr (D.-H.S.); 2 Sarcopenia Total Solution Center, Wonkwang University School of Medicine, Wonkwang University, 460 Iksandae-ro, Iksan 54538, Jeonbuk, Republic of Korea; sumin05@wku.ac.kr (S.-M.J.); kwjdragon0224@wku.ac.kr (W.-J.K.); 3 Department of Oriental Pharmacy, College of Pharmacy, Wonkwang University, 460 Iksandae-ro, Iksan 54538, Jeonbuk, Republic of Korea; 4 Department of Pharmacology, College of Korean Medicine, Kyung Hee University, Seoul 02447, Republic of Korea; thejinbong@khu.ac.kr; 5 Division of Pulmonary Medicine, Department of Internal Medicine, Wonkwang University School of Medicine, Iksan 54538, Jeonbuk, Republic of Korea; 6 Institute of Wonkwang Medical Science, Wonkwang University, 460 Iksandae-ro, Iksan 54538, Jeonbuk, Republic of Korea

**Keywords:** exosomes, inflammatory lung diseases, mesenchymal stem cells, plant-derived extracellular vesicles, antioxidant property

## Abstract

Inflammatory lung diseases are characterized by complex immune dysregulation and structural tissue damage, demanding the development of novel therapeutic and diagnostic strategies. Exosomes (Exos) have emerged as promising alternatives to address these challenges by serving as key mediators and effective therapeutic nanocarriers. This review systematically analyzes the multifunctional roles of Exos derived from various sources, including immune cells, mesenchymal stem cells (MSCs), lung structural cells, and non-mammalian sources such as plants and milk, in the context of inflammatory lung diseases. These vesicles modulate critical pathological processes, such as macrophage polarization, oxidative stress, and programmed cell death, by delivering functional cargos, including miRNAs and proteins. Studies demonstrating the antioxidant properties of Exos are classified, and their roles in attenuating oxidative stress-mediated lung injury are discussed. Furthermore, engineering and priming strategies, as well as airway-directed delivery methods such as nebulization, are reported to enhance therapeutic efficacy and targeting. Evidence also indicates that plant-derived Exos could be scalable and safer alternatives to mammalian cell-derived Exos. Collectively, Exos represent a next-generation platform for precision medicine, functioning as potent therapeutic agents and efficient drug-delivery systems for the treatment of complex inflammatory lung diseases.

## 1. Introduction

Lung inflammation is a fundamental pathological response to various insults, including infection, toxins, mechanical injury, and ischemic injury, and acts as a primary defense mechanism to preserve pulmonary homeostasis [1,2]. Acute inflammation is an immediate and adaptive response to infectious, chemical, or mechanical insults, aimed at eliminating pathogens, removing damaged tissue, and initiating repair [3]. In the context of acute lung injury (ALI) and acute respiratory distress syndrome (ARDS), however, excessive or uncontrolled inflammation precipitates diffuse alveolar damage, pulmonary edema, and severe hypoxemia, signifying a breakdown of the normal physiological balance that maintains alveolar–capillary integrity. Activated neutrophils and macrophages release large amounts of pro-inflammatory cytokines, proteases, and reactive oxygen species, which, while initially protective, can, when sustained, amplify tissue injury and compromise barrier function [4]. Especially, failure to resolve inflammation leads to dysregulated immune activation and inflammatory amplification in ALI and acute ARDS. When inflammatory injury persists, maladaptive repair mechanisms become dominant, with profibrotic signaling pathways, such as TGF-β/SMAD, promoting fibroblast activation and extracellular matrix deposition, ultimately predisposing the lung to chronic remodeling and fibrosis [5]. Although the chronic phase is pathologically destructive, the initial acute response is the decisive determinant of whether the lung undergoes successful repair or progresses to fibrosis [6]. Given the lack of effective therapies for chronic inflammatory diseases, early and precise control of inflammatory responses in ALI is critical not only for improving ALI-related mortality and morbidity but also for preventing inflammation-driven chronic lung diseases.

Despite continuous progress in critical care medicine, the treatment of ALI and ARDS remains largely supportive rather than curative. Adjunctive measures, including fluid restriction, prone positioning, extracorporeal membrane oxygenation (ECMO), and conservative oxygen therapy, aim to improve gas exchange and reduce secondary lung damage [7,8]. While they can temporarily stabilize respiratory function, they fail to prevent the progression from acute inflammation to irreversible alveolar damage and fibrosis. Pharmacological interventions—including corticosteroids, β-agonists, antioxidants, and anti-inflammatory agents—have yielded inconsistent results in clinical trials, often constrained by systemic side effects, narrow therapeutic windows, and limited efficacy in reducing mortality [9]. Furthermore, the complexity of ALI pathogenesis, involving immune dysregulation, oxidative stress, and endothelial–epithelial barrier dysfunction, presents major challenges for the development of targeted therapies. Current drugs lack the precision to modulate specific molecular pathways without disrupting essential host defense mechanisms. Collectively, these limitations underscore the urgent need for innovative therapeutic strategies to suppress excessive inflammation, protect the alveolar–capillary barrier, and promote endogenous repair mechanisms to restore pulmonary homeostasis.

Exosomes (Exos) are nanosized, lipid bilayer-enclosed extracellular vesicles (EVs) (30–150 nm in diameter) that originate from the endosomal pathway. Structurally, Exos contain a complex array of biomolecules, including proteins, lipids, metabolites, and nucleic acids such as mRNA, miRNA, and DNA fragments. Functionally, Exos mediate the transfer of bioactive molecules between cells, thereby influencing diverse biological processes, including immune modulation, tissue repair, angiogenesis, and inflammation [10]. Their lipid bilayer composition protects the internal cargo from enzymatic degradation, allowing stable circulation in various body fluids, including blood, urine, bronchoalveolar lavage fluid, and cerebrospinal fluid. Owing to these properties, Exos exhibit exceptional biocompatibility, low immunogenicity, and natural targeting capability toward recipient cells [11,12]. These advantages make Exos not only valuable as non-invasive diagnostic biomarkers but also as promising delivery vehicles for drugs, nucleic acids, and other therapeutic agents. Engineered or modified Exos can be tailored to enhance targeting specificity, increase cargo-loading efficiency, and improve therapeutic outcomes [13,14]. In this review, we summarize recent studies (primarily from the past three years) that highlight the therapeutic potential of Exos in ALI, a clinically relevant yet relatively underexplored model of acute inflammation compared to chronic pulmonary diseases. Notably, this manuscript provides a structured overview of Exos derived from various cell types, enabling comparison across studies, while also addressing their underlying therapeutic mechanisms and key technical considerations, such as isolation methods. In addition, balanced perspectives on limitations, potential adverse effects, and future directions are presented to enhance the translational relevance of this field.

## 2. Cell-Derived Exos

Cell-derived Exos originate from various mammalian cell types, including immune cells, mesenchymal stem cells (MSCs), and lung structural cells such as epithelial and endothelial cells. These vesicles are secreted directly by their parental cells and serve as natural mediators of intercellular communication, delivering proteins, lipids, and nucleic acids to target cells, thereby regulating immune responses, inflammation, and tissue repair [10]. In preclinical models of ALI and related respiratory disorders, unmodified or “native” cell-derived Exos have been shown to effectively suppress inflammatory cascades, modulate macrophage polarization, and restore epithelial–endothelial barrier integrity. Meanwhile, several studies have sought to enhance the therapeutic potential of these vesicles through priming strategies. Cytokine-stimulated Exos demonstrate superior anti-inflammatory, antioxidative, and reparative activities compared with non-primed Exos. These primed Exos often exhibit more potent tissue-protective effects than conventional, unmodified Exos [15]. In this section, we summarize and discuss the therapeutic effects of Exos derived from various sources in experimental models of lung injury.

### 2.1. Immune Cell-Derived Exos

Intercellular communication among immune cells plays a pivotal role in regulating inflammatory responses during ALI. Accumulating evidence indicates that EVs, particularly Exos, largely mediate this communication. These vesicles transfer diverse bioactive cargoes, including miRNAs, proteins, and lipids, between immune cell populations [16]. Following cellular uptake and subsequent activation of intracellular signaling pathways, immune cell-derived Exos have been shown to influence macrophage polarization, neutrophil migration and neutrophil extracellular trap (NET) formation, metabolic reprogramming, and cell survival. Preclinical evidence further supports the regulatory role of immune cell-derived Exos in ALI, as demonstrated by Exo administration. Several studies have demonstrated that macrophage-derived Exos exert protective effects in ALI models via diverse molecular mechanisms.

Ma et al. intraperitoneally administered macrophage-derived Exos and elucidated the EGFR–CXCR8–exo-miR-126a-3p axis as a key regulatory pathway in ALI, primarily through suppression of the PIK3R2/NLRP3 signaling cascade and attenuation of ferroptosis [17]. In parallel, intratracheal instillation models have highlighted the lung-targeted actions of macrophage-derived Exos. Yun et al. identified exosomal BMPR2 as a critical mediator of pulmonary repair, demonstrating that macrophage-derived Exos promote lung regeneration through specific molecular recognition and downstream signaling activation [18]. Similarly, Hu et al. reported that macrophage-derived exosomal TNF-α enhances pulmonary surfactant protein expression in PM2.5-induced ALI, demonstrating a context-dependent role of exosomal cytokine signaling in maintaining alveolar homeostasis [19]. Furthermore, Zheng et al. showed that RAW264.7-derived Exos protected against acute lung injury by inhibiting M1 macrophage activation and proliferation [20].

In addition to macrophage-mediated effects, regulatory mechanisms involving neutrophils have been reported. Jiao et al. demonstrated that intraperitoneally delivered M2 macrophage-derived Exos regulate polymorphonuclear neutrophil (PMN) migration and NET formation via lipid mediator class switching, thereby limiting PMN-mediated tissue damage in inflammatory lung injury [21].

Beyond naturally secreted immune cell-derived Exos, recent studies have extended this concept by engineering or cargo-loading Exo platforms to enhance immunoregulatory efficacy. FGF21-loaded M2 macrophage-derived Exos significantly protected against sepsis-induced lung injury by modulating inflammatory responses, macrophage polarization, cellular metabolism, and apoptosis [22]. Similarly, engineered mouse macrophage cell-derived Exos enriched with hsa-let-7i-5p exhibited immunomodulatory efficacy [23]. Not only for their therapeutic properties, some studies also use immune cell-derived Exos as drug-delivery carriers. Bao et al. reported that Exo derived from CD34^+^CD45^+^ cells alleviates ALI by enhancing macrophage efferocytosis [24]. Collectively, these findings indicate the importance of Exo-mediated immune cell crosstalk in immune homeostasis and tissue protection during inflammatory lung injury. Immune cell-derived Exos are listed in Table 1.

### 2.2. MSC-Derived Exos

MSCs, isolated from various tissue sources, are widely used as multipotent stromal cells with immunomodulatory and regenerative properties [25,26]. However, MSCs have several limitations, including phenotypic instability, vascular entrapment, immune rejection, and tumorigenic potential [27]. These safety concerns and practical limitations have driven growing interest in cell-free therapeutic strategies that retain the beneficial paracrine effects of MSCs while minimizing the risks associated with live-cell administration. Accumulating evidence indicates that the beneficial effects of MSCs are mediated by their secreted EVs, particularly Exos, which carry an array of bioactive molecules, including proteins, lipids, and regulatory RNAs [28]. Therefore, MSC-derived Exos have emerged as therapeutic alternatives that replicate key MSC functions while avoiding the limitations of live-cell transplantation [29]. In this review, we focused on Exos derived from these four representative MSCs, including bone marrow mesenchymal stem cells (BMSCs), adipose-derived mesenchymal stem cells (ADSCs), human umbilical cord mesenchymal stem cells (HUCMSCs), and placental mesenchymal stem cells (PMSCs), and summarized their therapeutic functions in ALI models (Table 2).

#### 2.2.1. BMSC-Exos

BMSC-Exos have been widely reported to attenuate lung injury through multiple immunoregulatory and cytoprotective mechanisms. Several studies have demonstrated that these Exos regulate immune cells through RNA cargo-mediated pathways. BMSC-derived exosomal miR regulates macrophage polarization in ALI models [30,31]. Not only for the macrophages, but BMSC-derived Exos also regulated neutrophil activation and NET formation [32]. In particular, exosomal miR-127-5p attenuates sepsis-associated ALI by inhibiting NET formation [33]. Beyond immune modulation, BMSC-Exos alleviate lung injury by regulating cell death-related pathways, particularly pyroptosis [34]. Although miRNA-related mechanisms have been most extensively reported, emerging evidence indicates that long noncoding RNAs (lncRNAs) loaded into Exos also contribute to the regulation of inflammatory responses [35].

In addition to native Exos, engineered or functionally modified BMSC-Exos further enhance therapeutic efficacy through targeted delivery and cargo optimization. Lin et al. demonstrated that mannose-modified BMSC-Exos improve macrophage-targeted delivery of miR-23b [36]. In addition to targeted delivery approaches, recent studies have shown that Exos can also be administered via nebulization, further supporting their broad therapeutic potential [37].

These Exos also exert significant antioxidant effects, contributing to their protective roles in inflammatory lung injury. These vesicles reduce oxidative stress-associated damage by modulating reactive oxygen species (ROS)-related signaling pathways and restoring cellular redox homeostasis. Several studies have reported that BMSC-Exos alleviate lung injury by suppressing inflammatory responses and oxidative stress. These protective effects have been consistently observed across diverse ALI models [38,39,40]. Collectively, these findings highlight the broad therapeutic potential of BMSC-Exos in inflammatory lung injury through coordinated immunomodulatory, cytoprotective, antioxidant, and tissue repair-associated mechanisms. The BMSC-Exos discussed in this section are summarized in Table 2, which includes detailed mechanistic insights.

**Table 2:** Therapeutic Roles of BMSC-Derived Exosomes.

Pathology	Exo Origin	Result	Administration	Isolation Method	Reference
ALI	BMSC-Exos	Regulation of LPS-induced macrophage polarization and alleviation of lung injury in ALI by MSC-derived exosomal miR-205-5p via the USP7/FOXM1 axis	Intravenous injection	Ultracentrifugation	[30]
	BMSC-Exos	Alleviation of lung injury and inflammation in ALI by BMSC-Exos via miR-137-3p-mediated M2 macrophage polarization	Intravenous injection	Ultracentrifugation	[31]
	BMSC-Exos	Attenuation of cardiopulmonary bypass-related ALI by BMSC-Exos via reduction in inflammatory response and oxidative stress	Intravenous injection	Ultracentrifugation	[40]
Sepsis-induced ALI	BMSC-Exos	Improvement of septic lung injury by BMSC-Exos via reduction in excessive NET formation and inflammatory response	Intraperitoneal injection	Ultracentrifugation	[32]
	BMSC-Exos	Alleviation of sepsis-related ALI by BMSC-derived exosomal miR-127-5p via inhibition of NET formation	Intratracheal instillation	Commercial Exo isolation kit	[33]
	BMSC-Exos	Inhibition of macrophage ferroptosis by BMSC-Exo-derived lncRNA SNHG12 for alleviating sepsis-induced lung injury	Intravenous injection	Ultracentrifugation	[35]
	Mannose-modified BMSC-Exos	Enhanced macrophage-targeted miR-23b delivery in sepsis-induced ALI via mannose functionalized BMSC-Exos	Intratracheal instillation	Ultracentrifugation	[36]
Ischemia/reperfusion injury	BMSC-Exos	Inhibition of CMPK2-mediated pyroptosis by BMSC-derived exosomal miR-202-5p for the alleviation of lung ischemia–reperfusion injury	Intravenous injection	Commercial Exo isolation kit	[34]
	BMSC-Exos	Alleviation of pulmonary ischemia/reperfusion injury by lncRNA-ZFAS1-carrying BMSC-Exos via UPF1-mediated FOXD1 mRNA decay	Intratracheal instillation	Commercial Exo isolation kit	[39]
Hemorrhagic shock-induced lung injury	BMSC-Exos	Inhibition of inflammation, oxidative stress, and apoptosis in HS-induced lung injury by HSF1-modified BMSC-Exos	Aerosol inhalation	Ultracentrifugation	[37]
Pneumonia-induced ALI	BMSC-Exos	Improvement in pulmonary gas exchange in pneumonia-induced ALI by BMSC-Exos	Intravenous injection	Ultracentrifugation	[38]

#### 2.2.2. ADSC-Exos

ADSC-Exos have emerged as important regulators of immune homeostasis and macrophage-associated inflammatory responses in ALI models, primarily by restoring alveolar macrophage homeostasis and modulating macrophage-derived cytokine signaling, such as TGF-β [41,42,43,44]. Several studies have suggested that ADSC-Exos regulate immune cell survival by modulating macrophage ferroptosis and pyroptosis through pathways such as SIRT1/NRF2 and miR-24-3p/NLRP3/Caspase-1/GSDMD, thereby suppressing excessive acute immune responses during ALI [45,46].

In addition to regulating immune cells, ADSC-Exos promote tissue repair in inflammatory lung injury. Mechanistically, several studies have highlighted the role of NRF2-related cytoprotective signaling in ADSC-derived Exo-mediated lung protection. Li et al. demonstrated that melatonin-stimulated MSC-derived exosomal LINC00052 activates the NRF2 pathway through the miR-152-3p/KLF4 axis to alleviate hyperoxia-induced lung injury [47]. In addition, Shen et al. showed that ADSC-derived exosomal miR-125b-5p alleviates pulmonary endothelial ferroptosis via the Keap1/NRF2/GPX4 pathway in sepsis-associated lung injury [48].

Beyond native Exos, modified or primed ADSC-Exos further enhance immunoregulatory efficacy. Exos derived from IFN-γ and TNF-α-primed ADSCs more effectively suppress lung injury [15,49]. Collectively, these findings support the therapeutic potential of ADSC-Exos in inflammatory lung injury by modulating macrophage-associated inflammatory responses and protecting lung tissues. ADSC-Exos included in this section are summarized in Table 3, with further mechanistic details.

#### 2.2.3. HUCMSC-Exos

HUCMSC-Exos have demonstrated therapeutic efficacy in inflammatory lung injury by modulating macrophage-mediated inflammatory signaling, regulating oxidative stress, and targeting cell death-associated pathways. Several studies have shown that HUCMSC-Exos regulate macrophage function through RNA-cargo-mediated mechanisms [50,51]. Especially, priming or preconditioning HUCMSC-Exos further enhances their cytoprotective and anti-inflammatory efficacy, particularly by attenuating oxidative stress-associated injury. IFN-γ-primed HUCMSC-Exos reduce oxidative stress and inflammatory responses in LPS-induced lung injury models [52]. Consistent with these findings, Bang et al. reported that thrombin-preconditioned HUCMSC-Exos significantly attenuate inflammation and tissue damage in bacterial-induced ALI models [53]. Collectively, these studies highlight the therapeutic relevance of HUCMSC-Exos in inflammatory lung injury by modulating macrophage-associated inflammatory responses, autophagy- and pyroptosis-related cell death pathways, and oxidative stress-associated cytoprotective signaling. The HUCMSC-Exos discussed in this section are summarized in Table 4, with additional mechanistic details.

#### 2.2.4. PMSC-Exos

PMSC-Exos have demonstrated protective effects in inflammatory lung injury by regulating inflammatory signaling, endothelial barrier integrity, and apoptosis-associated pathways. PMSC-Exos exhibit therapeutic efficacy comparable to their parental MSCs in inflammatory lung injury models. Valiukevičius et al. and Fang et al. both demonstrated that human PMSC-Exos effectively accelerate the recovery from lung injury. These treatments significantly reduce inflammation, attenuate oxidative stress-associated tissue injury, and improve alveolar barrier integrity, demonstrating efficacy comparable to direct MSC administration [54,55]. Several studies have also highlighted the role of PMSC-derived exosomal RNA cargo in mediating these lung-protective effects [56]. Antioxidant activity contributes substantially to the protective mechanisms of PMSC-Exos, as these vesicles can regulate redox homeostasis and suppress ROS-mediated cellular damage in the injured lung microenvironment [57]. Overall, these studies support the therapeutic relevance of PMSC-Exos in inflammatory lung injury. PMSC-Exos discussed in this section are summarized in Table 5, with further mechanistic insights.

### 2.3. Lung Structural Cell-Derived Exos

Endothelial cell-derived Exos have emerged as important regulators of immune cell crosstalk and inflammatory signaling in ALI [58].

Several studies have demonstrated that Exos released from pulmonary endothelial cells modulate macrophage polarization and inflammatory signaling through miRNA-mediated mechanisms. Chen et al. reported that aerosolized pulmonary microvascular endothelial cell (PMVEC)-derived Exos attenuate sepsis-induced ALI by modulating macrophage M1/M2 polarization through the heme oxygenase-1 (HO-1)–miR-184-3p–Sema7a signaling axis [59]. These studies highlight that airway-directed delivery enables localized immunomodulatory effects within the pulmonary microenvironment. In addition to endothelial cells, endothelial progenitor cell-derived Exos (EPC-Exos) have shown protective effects in lung injury models [60,61].

Exos derived from alveolar epithelial cells (AECs) represent another key signaling system involved in lung injury and repair. Intratracheal administration of Exos derived from type II AEC (AEC IIs) alleviates lung injury [62,63]. Similarly, human bronchial epithelial cell-derived EVs reduce the severity of lung injury [64]. These findings suggest that Exos derived from lung structural cells regulate ALI with airway delivery, enhancing localized immunomodulatory effects. Exos derived from lung structural cells, discussed in this section, are summarized in Table 6, along with detailed mechanistic insights.

## 3. Plant-Derived EVs and Milk-Derived Exos

Plant-derived EVs and milk-derived Exos have recently emerged as a distinct class of bioactive vesicles with therapeutic potential in inflammatory lung diseases. These vesicles can be mass-produced and delivered via non-invasive routes, such as oral administration, highlighting their promise as a therapeutic platform [65,66]. Milk-derived Exos provide early evidence for the feasibility of non-invasive vesicle-based interventions in lung injury. Filler et al. reported that gavage administration of bovine milk-derived Exos attenuated lung inflammation and injury in experimental necrotizing enterocolitis, demonstrating that orally delivered EVs can exert distal protective effects on the lung [67].

In parallel, plant-derived EVs have been shown to modulate macrophage metabolism and inflammatory responses in lung injury models. Fu et al. demonstrated that intravenously administered EVs derived from *Platycodon grandiflorum* attenuate lung injury by regulating macrophage inflammation and polarization through modulation of metabolic pathways, including lipid metabolism and glycolysis [68]. Beyond parenteral delivery, oral administration of plant-derived exosomal microRNAs has revealed additional gut-mediated mechanisms for lung protection. Specifically, Qiu et al. demonstrated that oral exosomal miR-7972 from fresh *Rehmanniae Radix* alleviated LPS-induced lung inflammation. This effect was achieved by modulating the GPR161–Hedgehog pathway and restoring gut microbiota balance, highlighting a potent gut–lung axis-mediated mechanism [69]. In addition to these naturally derived vesicle systems, recent studies have further enhanced therapeutic efficacy through Exo engineering strategies. Ma et al. developed neutrophil membrane-engineered EVs derived from *Panax ginseng* roots, loaded with miR-182-5p. Upon systemic administration, these engineered vesicles targeted the NOX4/Drp-1/NLRP3 signaling axis, markedly alleviating sepsis-induced ALI. This study illustrates how membrane cloaking and cargo optimization can significantly enhance the therapeutic efficacy of plant-derived EVs [70].

Taken together, these studies demonstrate that plant-derived EVs and milk-derived Exos represent emerging therapeutic carriers that modulate immune metabolism, inflammatory signaling, and the gut–lung axis in inflammatory lung injury. The plant-derived EVs and milk-derived Exos discussed in this section are summarized in Table 7, with detailed characterization.

## 4. Plasma-Derived Exos

The clinical application of miRNA-based therapeutics is limited by inefficient delivery and rapid degradation of free miRNAs in the circulation. Consequently, developing safe and efficient delivery vehicles is a critical challenge in advancing miRNA-based therapies [71]. In this context, Exos have emerged as promising endogenous nanocarriers that protect miRNAs from degradation and facilitate their functional delivery to recipient cells. Unlike synthetic loading strategies, Exos utilize endogenous sorting mechanisms to encapsulate and stabilize miRNA cargo. This natural process ensures physiologically relevant transfer while preserving the structural integrity of the vesicles [72]. Consistent with these findings, Yang et al. demonstrated that plasma-derived Exos naturally enriched in miR-124-3p effectively deliver this miRNA to macrophages, thereby promoting M2 polarization and alleviating ALI in a lung transplantation model. This study indicates that plasma-derived Exos function as biologically optimized nanocarriers that mediate miRNA-dependent immunoregulation, highlighting their potential clinical utility in inflammatory lung diseases [73]. The plasma-derived Exos discussed in this section are summarized in Table 8.

## 5. Discussion

Exos, important regulators of intercellular communication, are recognized as highly promising therapeutic and diagnostic tools for inflammatory lung diseases. These vesicles exert their biological functions by delivering cargo, including miRNAs, proteins, and lipids [74]. By delivering biological cargo, Exos regulate crucial physiological and pathological processes, including macrophage polarization, oxidative stress, epithelial barrier integrity, and programmed cell death pathways [75]. Therefore, Exos have gained research interest as promising intercellular mediators. Particularly in pulmonary diseases, circulating Exos in bronchoalveolar lavage fluid contain disease-specific molecules, including miRNAs, circRNAs, and inflammatory mediators, that reflect disease severity and progression [11,76]. Beyond their diagnostic value, Exos are recognized for their therapeutic potential to modulate various diseases. Moreover, Exos secreted by genetically modified cell lines could contain higher concentrations of specific biological cargo, suggesting their potential as therapeutic agents [77,78]. In this review, we summarized the functional roles of Exos derived from diverse sources, including immune cells, MSCs, lung structural cells, plant-derived vesicles, and circulating plasma, highlighting their involvement in modulating pulmonary inflammation, maintaining immune homeostasis, and promoting tissue repair.

MSC-derived Exos are the most extensively studied therapeutic platforms. MSCs can be isolated from various tissue sources, such as bone marrow, adipose tissue, umbilical cord, and placenta. Exos derived from these MSCs have been widely evaluated for their therapeutic potential in experimental ALI models. These findings indicate that Exos can replicate the therapeutic benefits of MSCs while avoiding the limitations of live-cell transplantation, including immune rejection, vascular entrapment, and tumorigenic risk, thereby supporting their potential as safer and more controllable therapeutic alternatives. Mechanistically, these Exos regulate macrophage polarization toward anti-inflammatory phenotypes, suppress pyroptosis and ferroptosis, reduce neutrophil NET formation, and restore epithelial–endothelial barrier integrity (Table 2, Table 3, Table 4 and Table 5). Importantly, recent studies demonstrate that engineered or primed MSC-derived Exos exhibit enhanced therapeutic efficacy through improved targeting efficiency [36] and optimized cargo composition [15]. In addition to MSC-derived vesicles, Exos from lung structural cells, including epithelial and endothelial cells, play a key role in pulmonary homeostasis and tissue repair [41]. Delivered Exos also regulate macrophage inflammatory signaling [51], metabolic reprogramming [63], and cell death pathways [62], underscoring their role as mediators of lung regeneration. Notably, not all immune cell-derived Exos exert beneficial effects. While macrophage-derived Exos have been extensively explored for their therapeutic potential, Exos from other immune cells, including neutrophils [79,80,81], CD4^+^ T cells [82], and mast cells [83], are more often implicated in exacerbating lung injury via pro-inflammatory signaling and cell death pathways. This distinction may explain why macrophage-derived Exos have been preferentially investigated as therapeutic candidates in inflammatory lung diseases. Beyond traditional immune-derived sources, telocytes, recognized as intercellular signaling hubs, have emerged as a potential source of therapeutic Exos [84]. Their Exos contributed to both tissue repair [85] and fundamental physiological processes [86], highlighting the need for broader investigation across diverse cell types.

Importantly, accumulating evidence suggests that the antioxidant properties of Exos represent a critical therapeutic mechanism in inflammatory lung diseases. Oxidative stress plays a central role in the pathogenesis of ALI and related pulmonary inflammatory disorders by promoting epithelial barrier disruption, amplifying inflammation, and inducing various forms of programmed cell death. As summarized in this review, several studies have demonstrated that Exos derived from MSCs and perinatal stem cells attenuate lung injury by reducing oxidative stress and restoring redox homeostasis. These antioxidant effects have been consistently observed across diverse experimental models, highlighting the ability of Exos to suppress oxidative stress-associated lung injury and inflammatory responses [37,40,52,57]. Taken together, these observations indicate that regulation of oxidative stress plays an important role in the protective effects of Exos. In ALI, oxidative stress is closely associated with inflammatory responses, mitochondrial dysfunction, and multiple forms of regulated cell death, suggesting that redox imbalance plays a central role in the progression of lung injury. Accordingly, modulation of oxidative stress via Exo-mediated cargo delivery offers a strategy to regulate several key pathological processes underlying lung injury. From a translational perspective, future studies should focus on developing Exos containing ROS-regulating cargos and identifying Exo sources with intrinsic antioxidant activity. These approaches support the development of Exo-based therapies to reduce oxidative damage and control inflammation in inflammatory lung diseases.

To translate these biological advantages into clinical practice, various strategies are being developed to optimize delivery and overcome the scalability limits of mammalian cell-derived Exos. From the perspective of therapeutic delivery for lung diseases, airway-directed modalities, such as nebulized aerosol inhalation, offer a targeted strategy to modulate the pulmonary microenvironment while minimizing off-target effects [87]. Recent evidence has demonstrated that nebulized Exo inhalation produces localized therapeutic effects, reduces systemic exposure, and enhances drug delivery to target lung tissues [88]. These advantages support the clinical applicability of inhalable Exos for the treatment of pulmonary inflammatory diseases. Furthermore, to address the challenges of mass production and the high costs associated with animal cell-derived Exos, non-mammalian cell-derived Exos have emerged as promising solutions. Plant-derived EVs represent a novel class of therapeutic nanocarriers, offering distinct advantages, including high scalability, low immunogenicity, and the potential for large-scale clinical application [89]. These vesicles have demonstrated the ability to modulate macrophage polarization [68], regulate inflammatory signaling [70], and influence gut–lung axis interactions [69], highlighting their unique therapeutic mechanisms. Furthermore, recent engineering approaches, including membrane functionalization and cargo loading, have significantly enhanced their targeting specificity and therapeutic efficacy [70]. These findings suggest that plant-derived vesicles could serve as a sustainable, scalable platform for Exo-based therapeutics, overcoming key limitations of mammalian cell-derived Exo production.

Despite their promising potential as novel therapeutics, several challenges have to be addressed before Exo-based therapies can be fully translated into clinical practice. One major limitation is the heterogeneity of Exos, as their composition and therapeutic efficacy depend on their cellular origin, physiological state, and isolation methods [90]. Accumulating evidence indicates that Exos could exert disease-promoting effects depending on their cellular origin and pathological context. In experimental ALI models, immune cell-derived Exos, particularly from neutrophils, CD4^+^ T cells, and mast cells, have been shown to exacerbate lung injury by promoting M1 macrophage polarization, pyroptosis, ferroptosis, oxidative stress, and inflammatory signaling [79,80,81,82,83]. In addition, fibroblast-derived Exos have been implicated in profibrotic processes, with specific cargoes correlating with the severity of interstitial fibrosis and the presence of fibrotic EV phenotypes in patient-derived samples [91]. These findings highlight that Exos are functionally heterogeneous and may contribute to disease progression, underscoring the need for careful source selection and rigorous quality control in therapeutic applications. At the same time, emerging clinical evidence supports the therapeutic and diagnostic potential of Exo-based approaches. Recent studies have demonstrated the therapeutic efficacy of Exo-based treatments in severe ALI, including COVID-19-associated cases [92,93], and circulating exosomal miRNAs, such as miR-21, miR-155, and miR-146a, are being explored as clinical biomarkers to assess disease severity in ALI [94]. Taken together, these findings highlight both the promise and the complexity of Exo-based applications, emphasizing the need for standardized protocols for production, purification, and characterization to ensure reproducibility and clinical safety.

To ensure the successful clinical translation of MSC-Exos, researchers are implementing standardized manufacturing protocols and rigorous quality control matrices in accordance with GMP and EMA guidelines. This framework focuses on validating parental cell integrity by demonstrating high viability (>90%) and confirming the final product’s molecular identity using exosomal protein signatures such as CD63 and CD81. Additionally, stringent microbiological assessments, including endotoxin and sterility testing, are mandatory to ensure the safety and therapeutic potency of the Exo-based therapeutic [95]. Furthermore, the biodistribution, pharmacokinetics, and long-term safety of Exo-based therapies require comprehensive evaluation. Addressing these challenges will require establishing GMP-compliant production systems and standardized quality control frameworks. Also, future research should focus on advancing Exo engineering technologies to improve targeting specificity, cargo-loading efficiency, and therapeutic potency. Surface modification strategies, genetic engineering of donor cells, and development of inhalable or aerosolized Exo formulations could significantly improve therapeutic efficacy and clinical approach. In addition, integrating multi-omics profiling with artificial intelligence-based analysis will enable the identification of novel therapeutic targets and facilitate the development of precision Exo therapeutics. In addition, emerging technologies such as organ-on-chip systems, human organoids, and artificial intelligence-based platforms are increasingly being integrated into Exo research to enable more physiologically relevant disease modeling and precision therapeutic evaluation [96]. These approaches offer significant potential to improve the predictive accuracy of preclinical studies and to facilitate precision clinical translation [97]. However, several technical challenges remain, including model standardization, scalability, and the integration of multi-modal data, which must be addressed to fully realize their clinical utility. In this context, as discussed in Section 2.1, further engineering of exosomes by incorporating functional cargoes such as miRNAs may enable the development of advanced, next-generation therapeutic platforms.

## 6. Conclusions

Exos are multifunctional biological nanocarriers with considerable potential as therapeutic agents, drug-delivery vehicles, and diagnostic biomarkers for ALI and related inflammatory lung diseases. While their therapeutic benefits are well established, recent evidence suggests that Exos can also promote disease, depending on their cellular origin and the specific disease context. This functional heterogeneity emphasizes the necessity for careful source selection, rigorous quality control, and standardized production protocols to ensure safety and reproducibility. Advances in Exo engineering, scalable manufacturing, and targeted delivery technologies are likely to accelerate clinical translation. Additionally, integrating multi-omics profiling and precision medicine strategies will support the identification of optimal Exo populations and therapeutic cargos. Ongoing progress in these technological and translational advances is expected to establish Exo-based therapeutics as a foundational platform for next-generation treatments of complex pulmonary diseases.

## Figures and Tables

**Table 1:** Therapeutic Roles of Immune Cell-Derived Exosomes.

Pathology	Exo Origin	Result	Administration	Isolation Method	Reference
ALI	Macrophage-derived Exos	Inhibition of PIK3R2/NLRP3 signaling and ferroptosis by EGFR/CXCR8–exo-miR-126a-3p in ALI	Intraperitoneal injection	Commercial Exo isolation kit	[17]
	Macrophage-derived Exos	Pulmonary repair in ALI via molecular recognition and signaling activation mediated by BMPR2-loaded Exos	Intratracheal instillation	Ultracentrifugation	[18]
	Macrophage-derived Exos	Enhanced pulmonary surfactant protein expression in PM2.5-induced ALI mediated by macrophage-derived exosomal TNF-α	Intratracheal instillation	Ultracentrifugation	[19]
	CD34^+^CD45^+^ cell-derived Exos	Enhancement of macrophage efferocytosis and therapeutic recovery in ALI through CD34^+^CD45^+^ cell-derived Exos	In vitro	Ultracentrifugation	[24]
Sepsis-induced ALI	RAW264.7 murine macrophage-like cell-derived Exos	Repeated administration of Exos enabled sustained protection against sepsis-induced ALI by enhancing resistance to subsequent LPS rechallenge	Intravenous injection	Ultracentrifugation	[20]
	M2 macrophage-derived Exos	Regulation of PMN migration and NET formation in sepsis-induced ALI by M2-Exos via lipid mediator class switching	Intraperitoneal injection	Commercial Exo isolation kit	[21]
	FGF21-loaded M2 macrophage-derived Exos	Regulation of inflammatory, metabolic, and apoptotic pathways in septic lung injury by FGF21-M2-Exo	Intratracheal instillation	Ultracentrifugation	[22]
	hsa-let-7i-5p-engineered RAW264.7 Exos	Therapeutic efficacy in endotoxemic sepsis mediated by hsa-let-7i-5p-enriched engineered Exos derived from RAW264.7 cells	Intraperitoneal injection	Ultracentrifugation	[23]

**Table 3:** Therapeutic Roles of ADSC-Derived Exosomes.

Pathology	Exo Origin	Result	Administration	Isolation Method	Reference
ALI	ADSC-Exos	Alleviation of HILI by melatonin-stimulated ADSC-Exosomal LINC00052 via the miR-152-3p/KLF4/Nrf2 axis	Intravenous injection	Commercial Exo isolation kit	[47]
	Exos from IFN-γ and TNF-α-primed ADSC	Robust attenuation of inflammatory cytokines, immune cell recruitment, and lung injury markers in ALI by primed ADSC-Exos in LPS-challenged mice	Intravenous injection	Tangential Flow Filtration (TFF system)	[15]
	Exos from IFN-γ and TNF-α-primed ADSC	Modulation of experimental ALI by MSC-derived exosomal miR-7704 via M2 macrophage polarization	Intratracheal instillation	Ultracentrifugation & Commercial Exo isolation kit	[49]
Sepsis-induced ALI	ADSC-Exos	Restoration of cellular homeostasis and immune balance by ADSC-Exos in sepsis-induced lung injury via modulation of intercellular communication	Intravenous injection	Commercial Exo isolation kit	[41]
	ADSC-Exos	Restoration of alveolar macrophage homeostasis and modulation of immune responses in sepsis-induced ALI by ADSC-Exos	Intratracheal instillation	Ultracentrifugation	[42]
	ADSC-Exos	Attenuation of sepsis-induced ALI and inflammatory responses by ADSC-Exos via induction of macrophage TGF-β secretion in CLP mice	Intravenous injection	Ultracentrifugation	[43]
	ADSC-Exos	Suppression of macrophage ferroptosis by ADSC-Exos via the SIRT1/NRF2 axis to alleviate sepsis-induced ALI	Intravenous injection	Ultracentrifugation	[45]
	ADSC-Exos	Attenuation of sepsis-induced ALI by ADSC-Exos through modulation of macrophage pyroptosis via the miR-24-3p/NLRP3/Caspase-1/GSDMD axis	Intravenous injection	Ultracentrifugation	[46]
	ADSC-Exos	Inhibition of ferroptosis in sepsis-induced ALI by ADSC-Exos through the Keap1/Nrf2/GPX4 pathway	Intravenous injection	Ultracentrifugation	[48]
SARS-CoV-2 and H1N1 influenza-induced ALI	ADSC-Exos	Alleviation of virus-infection-associated ALI using ADSC-Exos	Intravenous injection	Tangential Flow Filtration (TFF system)	[44]

**Table 4:** Therapeutic Roles of HUCMSC-Derived Exosomes.

Pathology	Exo Origin	Result	Administration	Isolation Method	Reference
ALI	HUCMSC-Exos	Improvement of ALI by HUCMSC-exosomal miR-451 via suppression of alveolar macrophage autophagy through the TSC1/mTOR pathway	Intravenous injection	Commercial Exo isolation kit	[50]
	HUCMSC-Exos	Alleviation of ALI by HUCMSC-Exos via inhibition of alveolar macrophage pyroptosis	Intratracheal instillation	Ultracentrifugation	[51]
	IFN-γ-primed HUCMSC-Exos	Reduction in oxidative stress and inflammatory responses in ALI by IFN-γ-primed HUCMSC-Exos	Intravenous injection	Ultrafiltration tube & Ultracentrifugation	[52]
*Escherichia coli*-induced ALI	Thrombin-preconditioned HUCMSC-Exos	Significant reduction in *E. coli*-induced inflammation and lung tissue damage by intratracheal administration of thrombin-preconditioned HUCMSC-Exos	Intratracheal instillation	Tangential Flow Filtration (TFF system)	[53]

**Table 5:** Therapeutic Roles of PMSC-Derived Exosomes.

Pathology	Exo Origin	Result	Administration	Isolation Method	Reference
ALI	PMSC-Exos	Enhanced resolution of ALI by PMSC-Exos through the attenuation of inflammation and restoration of alveolar barrier integrity	Intraperitoneal injection	Tangential Flow Filtration (TFF system)	[54]
	PMSC-Exos	Improvement of lung function and reduction in inflammation in ALI by PMSC-Exos	Intravenous injection	Ultracentrifugation	[55]
	PMSC-Exos	Protection against endothelial barrier disruption of HPVECs by PMSC-Exos in ALI/ARDS via the hsa-miR-148a-3p/ROCK1 signaling pathway	Not provided	Commercial Exo isolation kit	[56]
Aspiration Pneumonia-induced ALI	PMSC-Exos	Mitigation of ALI by intratracheal administration of HUCMSC-Exos via hsa-let-7i-5p-mediated modulation of inflammation, oxidation, and apoptosis in a murine AP model	Intratracheal instillation	Ultracentrifugation	[57]

**Table 6:** Lung structural cell-derived Exos (endothelial + epithelial).

Pathology	Exo Origin	Result	Administration	Isolation Method	Reference
ALI	EPC-Exos	Protection against macrophage inflammation in ARDS by EPC-exosomal miR-103-3p via HnRNPA2B1-mediated delivery and inactivation of the TLR4/NF-κB pathway	Intratracheal instillation	Ultracentrifugation	[60]
	Exos derived from AEC IIs	Inhibition of the necroptosis pathway in ALI by AECII-derived exosomal miR-21a-5p via modulation of PGAM5	Intratracheal instillation	Commercial Exo isolation kit	[62]
	Exosomal STIMATE derived from AEC IIs	Control of metabolic reprogramming in tissue-resident alveolar macrophages in ALI by AECII-derived exosomal STIMATE	Aerosol inhalation	Ultracentrifugation	[63]
	Human bronchial epithelial cell-derived EVs	Attenuation of ALI and inflammatory responses through intratracheal delivery of HBEC-derived EVs	Intratracheal instillation	Ultracentrifugation	[64]
Sepsis-induced ALI	Exos derived from PMVECs	Alleviation of sepsis-induced ALI by HO-1 via modulation of the M1/M2 macrophage ratio through regulation of the miR-184-3p/Sema7a axis	Aerosol inhalation	Ultracentrifugation	[59]
	EPC-Exos	Mitigation of septic ALI by EPC-exosomal miR-218 via inhibition of HMGA1-mediated macrophage polarization	Intravenous injection	Ultracentrifugation	[61]

**Table 7:** Therapeutic Roles of Plant- and Milk-Derived Extracellular Vesicles.

Pathology	Exo Origin	Result	Administration	Isolation Method	Reference
ALI	*Platycodon grandiflorum* Exo-like nanoparticles	Regulation of macrophage inflammation and polarization via metabolic reprogramming by PGLNs to alleviate ALI	Intravenous injection	Sucrose density gradient with ultracentrifugation	[68]
	Plant exosomal miRNA derived from fresh *Rehmanniae Radix* (Rgl-exomiR-7972)	Alleviation of LPS-induced ALI and gut dysbiosis by *Rehmanniae Radix*-derived miR-7972 via the GPR161/Hedgehog axis	Oral gavage	Sucrose density gradient with ultracentrifugation	[69]
Sepsis-induced ALI	Neutrophil membrane-engineered *Panax ginseng* root-derived Exos	Alleviation of sepsis-induced ALI by neutrophil membrane-engineered *Panax ginseng* Exos via the miRNA-182-5p/NOX4/Drp-1/NLRP3 axis	Intravenous injection	Sucrose density gradient with ultracentrifugation	[70]
Secondary ALI induced by necrotizing enterocolitis	Bovine milk-derived Exos	Attenuation of NEC-induced lung inflammation and injury by bovine milk-derived Exos	Oral gavage	Ultracentrifugation	[67]

**Table 8:** Therapeutic Roles of Plasma-Derived Exosomes.

Pathology	Exo Origin	Result	Administration	Isolation Method	Reference
ALI	Plasma-derived Exos	Regulation of macrophage polarization and ALI in lung transplant recipients by miR-124-3p	Intravenous injection	Commercial Exo isolation kit	[73]

## Data Availability

No new data were created or analyzed in this study. Data sharing is not applicable to this article.

## Abbreviations

The following abbreviations are used in this manuscript:

Exo	Exosome
ALI	Acute lung injury
ARDS	Acute respiratory distress syndrome
ECMO	Extracorporeal membrane oxygenation
EVs	Extracellular vesicles
MSC	Mesenchymal stem cell
BMSCs	Bone marrow mesenchymal stem cells
ADSCs	Adipose-derived mesenchymal stem cells
HUCMSCs	Human umbilical cord mesenchymal stem cells
PMSCs	Placental mesenchymal stem cells
NET	Neutrophil extracellular trap
PMN	Polymorphonuclear neutrophil
PMVECs	Pulmonary microvascular endothelial cells
HO-1	Heme oxygenase-1
EPC-Exos	Endothelial progenitor cell-derived exosomes
AECs	Alveolar epithelial cells
AEC IIs	Type II alveolar epithelial cells
